# Preparation of Nitrogen and Phosphorus Doped Porous Carbon from Watermelon Peel as Supercapacitor Electrode Material

**DOI:** 10.3390/mi14051003

**Published:** 2023-05-06

**Authors:** Chi Yang, Penghui Li, Yumeng Wei, Yanting Wang, Bo Jiang, Wenjuan Wu

**Affiliations:** 1 College of Light Industry and Food Engineering, Nanjing Forestry University, Nanjing 210037, China; flipped@njfu.edu.cn (C.Y.); liph@njfu.edu.cn (P.L.); yyqx@njfu.edu.cn (Y.W.); ytw@njfu.edu.cn (Y.W.); bjiang@njfu.edu.cn (B.J.); 2 Jiangsu Co-Innovation Center of Efficient Processing and Utilization of Forest Resources, Nanjing Forestry University, Nanjing 210037, China

**Keywords:** watermelon peel, porous carbon, supercapacitors, electrode material

## Abstract

The use of green and sustainable biomass-derived compounds to obtain excellent electrochemical properties is important to address growing environmental and energy issues. In this paper, cheap and abundant watermelon peel was used as a raw material to successfully synthesize nitrogen-phosphorus double-doped bio-based porous carbon by a one-step carbonization method and explore it as a renewable carbon source for low-cost energy storage devices. The supercapacitor electrode exhibited a high specific capacity of 135.2 F/g at a current density of 1 A/g in a three-electrode system. A variety of characterization methods and electrochemical tests indicate that porous carbon prepared by this simple method has great potential as electrode materials for supercapacitors.

## 1. Introduction

With the development of society, the global energy demand is increasing, fossil fuel consumption will cause serious damage to the ecological environment, and the research and utilization of storage devices using renewable energy is urgently needed to solve the problem [1]. Supercapacitors have been highlighted in the field of electrochemical applications because of their properties such as environmental friendliness, excellent cyclic performance, and high power [2]. It has been applied as a new kind of energy storage device in the field of power systems and other fields with great prospect [3]. The selection of electrode material plays a decisive role in the performance of supercapacitor; therefore, the development of electrode materials with large specific surface area, high conductivity, high specific capacitance, and low resistance can increase the electrolyte ions accommodated in supercapacitors, thus enhancing the performance [4]. All kinds of carbon are used as electrode materials for supercapacitors: activated carbon accounts for most of them [5], and then there’s graphene [6], carbon nanotubes [7], their composites [8], etc. Activated carbon has good electric conductivity, high specific surface area and physicochemical stability, and has been brought to universal application as electrode material in electrochemical double-layer capacitors [9]. It has been shown that doping the electrode material with heteroatoms leads to another significant improvement in the electrochemical performance of the capacitor. For example, nitrogen doping creates defects in the material that convert inert graphene-like layered carbon into electrochemically active material without affecting its conductivity, resulting in a significant increase in capacitance of supercapacitors and the ability to bipolarly charge or discharge at a fast rate [10]. Phosphorus doping results in a significant increase in the theoretical capacity, specific surface area, and charge mobility of the electrode material [11]. Heteroatom doping effectively enhances the electrochemical properties of carbon materials, making them promising electrode materials for energy storage devices. Although commonly used carbon materials have decent capacitance, synthesizing them can cause serious environmental pollution and high cost, and these carbon materials usually come from progressively depleting non-renewable resources. As a consequence, the search for renewable resources that can be used as carbon materials is urgently needed. For the last few years, carbon materials derived from biomass and its residue have been widely researched because of their perfect cost-effectiveness, high resourcefulness, environmental compatibility, and excellent performance. Rationalizing the use of biomass material residue to prepare porous carbon supercapacitor electrodes can achieve industrial production for high-performance supercapacitor electrodes while realizing the utilization of biomass waste, which has great potential [12].

Biomass-derived carbonaceous materials can be used to replace the current petrochemical industry by converting them into cost-effective and excellent electrochemical performed devices [13]. Biomass is environmentally friendly, has a large volume and wide distribution, and is widely derived from agriculture, forestry, and municipal waste [14,15,16]. Additionally, it can be converted into green energy and industrial production materials [17]. The diversity of biomass species and various processing methods make its structural enrichment and wide range of applications possible [18]. Carbon materials have special structures and excellent properties. Moreover, the carbon material obtained from biomass conversion possesses high determination of carbon and reproducible characteristics, and the use of bio-based feedstock to produce a variety of carbon materials can reduce production costs and also achieve sustainable development of carbon materials [19]. Because they are produced in an environmentally friendly way with objective benefits, in large scale, and in a simple and controlled manner, the application of biomass-derived carbonaceous materials based on various pathways is widely explored. The conversion of biomass to carbon nanomaterials shows promising prospects to add value to biomass compared to the conventional bio-derived synthesis hydrates, biochar, and activated carbon [20]. Biomass resources are considered on the list of the most prospective sustainable raw materials to replace fossil resources. However, the valorization of biomass still has a huge scope for exploration [21]. The preparation of biomass-based electrode materials has become a recent hotspot due to the simple manufacturing method, low cost, renewability, and abundance [22]. For example, waste materials from fruits such as oranges [23], watermelons [24], blueberries [25], and cherries [26] are applied as electrode materials in supercapacitors by carbonization and other methods.

Notably, watermelon, a worldwide prevalent fruit, accounts for 7% of total fruit and vegetable production [27]. Its peel accounts for about 40% of the whole weight of the watermelon [28]. However, the watermelon peel is often considered as agricultural waste, leading to biomass loss. Currently, watermelon peels are mostly sent directly to landfills, combusted, applied to animal feedstuff, or composted, with little added value treatments, and even have an additional negative environmental impact [29]. In order to increase its added value, the application prospects of watermelon peel are being explored in a number of ways, including as an adsorbent material [30], preparation of catalysts [31,32], etc. Over the past few decades, electrode materials prepared by carbonization and other means using watermelon peel as a raw material have been gradually used in supercapacitors. Omar et al. [33] developed a new binary composite material based on watermelon peels to be used as a supercapacitor electrode with excellent properties of nitrogen enrichment and high stability. Zhang et al. [34] used watermelon peel-derived heteroatom-doped multistage porous carbon as a high-performance electrode material for supercapacitors, exhibiting excellent electrochemical performance. Tu et al. [35] synthesized watermelon peel-derived carbon aerogel by a facile green procedure and used it as an electrode material to make supercapacitors, and the test results showed that the supercapacitors had fast charging and discharging capability. Consequently, the waste part of the watermelon is available as a prospective biomass resource to prepare porous biocarbon as an electrode material for efficient and environmentally friendly electrochemical supercapacitors [36]. Here in, we used waste watermelon peel as a carbon source and nitrogen source, phosphoric acid as phosphorus source doping, and used Muffle furnace carbonization one-step process to prepare carbon materials—Muffle furnace watermelon peel carbon material (MWC).

## 2. Materials and Methods

### 2.1. Source of Materials

Watermelon peel was derived from waste materials from a fruit market in Nanjing, Jiangsu, China. It was dried in a blast dryer at 105 °C for 72 h and crushed into 50–80 meshes.

Sinopharm Chemical Holding Co., Ltd. (Shanghai, China) provided the chemicals for phosphoric acid (density 1.69), conductive carbon black, and poly tetra fluoroethylene (PVDF). The chemicals were not further purified. Deionized water was prepared in the laboratory.

### 2.2. Preparation Method

**Figure 1:**
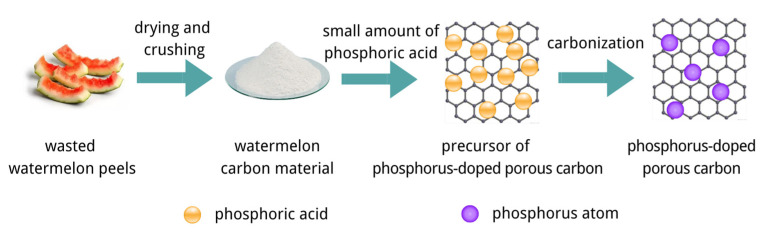
Preparation method of nitrogen and phosphorus doped MWC material.

The porous carbon doped with phosphorus was synthesized. A total of 10 g of waste watermelon peel powder was mixed with 12 mL phosphoric acid, and the mass ratio of watermelon peel powder to phosphoric acid was 1:2. Subsequently, the samples were subjected to a carbonization process using a muffle furnace by heating them in air at a heating rate of 700 °C to 10 °C and holding them at 700 °C for 2 h. Once cooled down to the indoor temperature, the crucible was removed from the Muffle furnace. The product was then washed several times in deionized water and dried for 6 h in a 105 °C oven. The preparation of nitrogen and phosphorus doped porous carbon from watermelon peel is illustrated in Figure 1.

## 3. Analytical Method

### 3.1. Scanning Electron Microscope Test Analysis (SEM)

The surface morphology of our fabricated MWC was observed. The instrument was from Hitachi, Tokyo, Japan, model Quanta 20 field emission scanning electron microscope. Before the test, the carbon material was adsorbed on the conductive adhesive surface of the observation platform and then treated with gold spray so as to avoid static electricity.

### 3.2. X-ray Photoelectron Spectroscopy

X-ray photoelectron spectroscopy was used to observe the surface chemical composition of watermelon carbon materials and to determine the details of peaks, such as the peak of C(C 1s), N(N 1s), and P(P 2p). The instrument used was manufactured by Shimadzu Company of Kyoto, Japan, model AXIS Ultra DLD X-ray photoelectron spectrometer. More than two groups of watermelon carbon materials were prepared for determination with a depth of investigation controlled within 10 nm, and more than three different locations were replaced each time. This provided information on the elemental composition and content, chemical state, molecular structure, and chemical bonding of various compounds for the study of electronic materials. The deconvolution of the XPS peaks were performed by a Shirley background subtraction, followed by peak fitting with Voigt functions with mixed Gaussian and Lorentzian features [37].

### 3.3. X-ray Diffraction Spectrum Analysis

X-ray diffraction spectroscopy was used to observe the crystal structure of the carbon material of watermelon using an instrument from Tokyo, Japan—model Nikko Ultima IV combined multifunctional horizontal X-ray diffractometer, using a CuKα target (λ = 0.15406 nm) as the incident source. For removing the effect of CuKα radiation, a graphite monochromator was used in 40 kV X-ray tube voltage, 200 mA X-ray tube current, and a 2θ = 5~60° range of scanning. During the scanning process, the system step was set to 0.02° and the speed was 15°/min. According to the experimental results, the “diffraction intensity-2θ” curve was plotted.

### 3.4. Thermogravimetric Analysis

In order to observe the thermal stability of our watermelon carbon material, the thermogravimetric analyzer, the model TGA209 F1 thermogravimetric analyzer, was used to test the analytical instrument. The instrument was from Bavaria, German. The thermogravimetric analyzer was protected by high-purity nitrogen and was heated at the rate of 10 °C /min in the temperature range of 30–750 °C.

### 3.5. Electrochemical Performance Test

#### 3.5.1. Preparation of Working Electrode

Firstly, poly tetra fluoroethylene (PTFE), conductive carbon black, and MWC electrode materials were prepared in the mass ratio of 1:1:8. Then, rind was put in a mortar to make the material above fully mixed. A total of 12–15 drops of N-methyl pyrrolidone were dropped into it and a 15 min ultrasonic shock was carried out to make it more fully mixed. A total of 5.2 mg of it was applied on nickel foam, evenly coated. The nickel foam was put in a vacuum oven and the drying temperature of 80 °C and drying time of 12 h was controlled to get the required working electrode.

#### 3.5.2. Test Method

For observing the electrochemical properties of MWC materials, the cyclic voltammetry curve (CV) and galvanostatic charge–discharge curve (GCD) were measured and analyzed by Shanghai Chenhua CHI660E electrochemical workstation. The test environment was 6 mol/L KOH electrolyte solution. Platinum plate electrode was used as the counter electrode and Ag/AgCl electrode was used as the reference electrode. The control voltage was within the range of 0.5 V to 1 V, the CV of the working electrode was obtained at the various scan rates (10–50 mV/s) using 3-electrode setup, the control voltage ranged from −0.2 V to 0.8 V, and the GCD was gained. The current densities under different test conditions were, respectively, 1, 3, 5, 10, 20, and 30 A/g. The specific capacitance of the electrode material was calculated by Equation (1) at an amplitude of 5 mV under the condition that the initial voltage was open-circuit voltage, electrochemical impedance spectra was carried out on the working electrode, and the control frequency was within the range of 10^−2^–10^5^ Hz to detect its cycle service life. The galvanostatic charge/discharge test was repeated at the current density of 1 A/g 1000 times.

Equation (1) is a formula of a linear charge–discharge curve:

(1) $$C_{m}=C/m=I\Delta t/\left(m\Delta v\right)$$

where *C_m_*(F/g) indicates its specific capacitance; *m*(g) indicates the loading of the corresponding electrode material; *I*(A) indicates the charging/discharging current; Δ*t*(s) indicates the charging/discharging duration; Δ*v*(V) indicates the voltage window.

Equation (2) is a formula of a nonlinear charge–discharge curve:

(2) $$C_{m}=\frac{2\times I\times S}{m\times \Delta U^{2}}=\frac{2\times I\int_{t\left(U_{\max}\right)}^{t\left(U_{\min}\right)}U\left(t\right)dt}{m\times \Delta U^{2}}$$

where *I*(A) indicates charging/discharging current; *S*(V_s_) indicates the integration area beneath the discharging curves of the electrode material; *m*(g) indicates the mass of the active substance; *t*(*U_max_*) indicates the starting duration of discharge (s); *t*(*U_min_*) indicates the closing duration of discharge (s); Δ*U*(V) indicates its voltage swing at which the voltage decay is discounted.

## 4. Results and Discussion

### 4.1. Morphology and Structure of MWC

**Figure 2:**
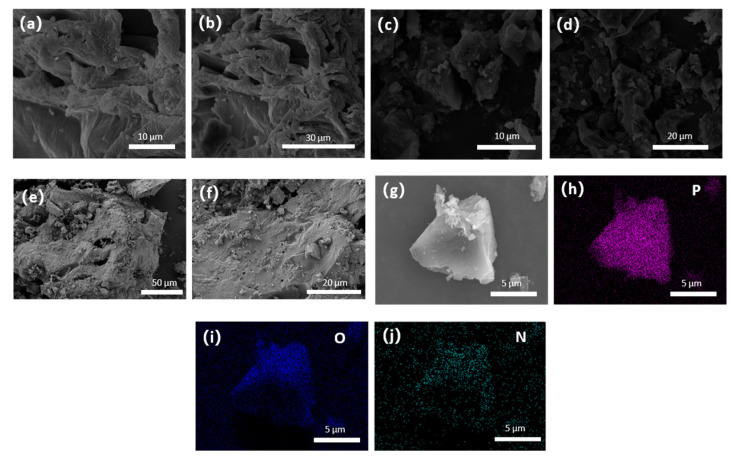
SEM images of (**a**,**b**) watermelon peel, (**c**,**d**,**g**) MWC, (**e**,**f**) MWC after 1000 charge/discharge cycles under a current load of 1 A/g; EDS element map of MWC, (**h**) P element, (**i**) O element, (**j**) N element.

The surface morphology of watermelon peel and watermelon peel carbon material was observed by SEM, and it was observed that there were obvious morphological differences before and after carbonization. As shown in Figure 2a,b, watermelon peel before carbonization has a lamellar shape and a rough surface, showing a connected structure. After carbonization (Figure 2c,d), the carbon matrix of watermelon peel is more dispersed and smaller, with some pores. The increase of porosity is beneficial to increase its specific surface area, promote electrolytic solution infiltration and carrier transmission, improve the capacitance, as well as rate performance of watermelon peel carbon material, and enhance its electrical conductivity [38]. SEM images show a relatively collapsed carbon matrix with a small amount of irregular broken surface, which may be caused by oxidation in the Muffle furnace [39]. Under SEM, the manufactured MWC looked similar to the raw material, indicating that sulfur was well incorporated in it. Phosphoric acid doping may facilitate ion and electron conduction and ultimately improve electrochemical performance [40]. In addition, as demonstrated in Figure 2e,f, in order to test the stability, SEM was used to observe the surface morphology of MWC material after 1000 times of charging and discharging under a current of 1 A/g. The results showed that the porosity of the material increased, indicating that MWC has good conductivity and stability. The EDS elemental mapping image in Figure 2e, f shows P, N, and O elements, demonstrating the successful doping of N and P elements in biomass carbon.

**Figure 3:**
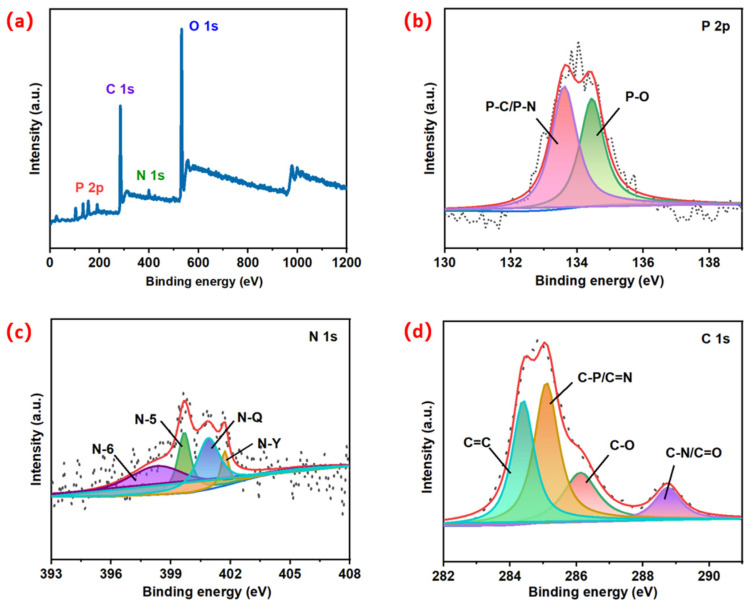
(**a**) Full scan spectra of MWC derived using XPS and deconvoluted (**b**) P 2p spectra, (**c**) N 1s spectra, (**d**) C 1s spectra of MWC.

Further analysis of the element content and bonding state of MWC was carried out using XPS. As illustrated in Figure 3, the total scan spectrum of MWC could be fitted into four peaks; among them, the double peaks of 284.5 eV and 531.5 eV are typical of the coexistence of C 1s and O 1s [41]. In addition, the full scan spectra exhibited two distinct peaks at approximately 133.5 eV and 399.5 eV, corresponding to P 2p (phosphorus) and N 1s (nitrogen), confirming the presence of heteroatoms, as shown in Figure 3b. Further deconvolution of P 2p peaks for phosphorus yields two peaks with binding capacities of 133.6 eV and 134.4 eV, corresponding to P-C/P-N and P-O bond, respectively, which reveals the successful doping of phosphorus, provides more electrochemical active sites and higher wettability, and promotes the interaction between electrolyte and battery, thus improving the electrochemical performance of the material [42]. The XPS N 1s map of MWC in Figure 3c can also be deconvolved into four peaks, which correspond to N-6 (pyridine N, 398.3 eV), N-5 (pyrrole N, 399.7 eV), N-Q (graphite N, 400.9 eV), as well as N-Y (nitrogen oxide bond, 401.7 eV). The existence of carbon atoms of pyrrole and pyridine was further confirmed [43]. The results of related studies have demonstrated that pyrrole N and pyridine N can be used as faradic reactive sites to provide pseudocapacitors and improve capacitive performance. The insertion of N-Q allows electrons to move more easily in the carbon lattice and increase specific capacitance [44]. As illustrated in the MWC C 6s (Figure 3d), four types of peaks can be seen at 284.4, 285.1, 286.1, and 288.7 eV, which correspond to C=C, C−P/C=N, C−O, and C=O/C−N, individually. These bonds’ formation is conducive to enhance the electrochemical REDOX activity and wettability of the material. It promotes the mutual action among electrode materials and electrolyte solutions [45]. The doped N and P heteroatoms contribute pseudocapacitors to the charging/discharge process while improving the hydrophilicity of the surface of carbon materials. In particular, the surface of MWC presents an objective content of pyridine N, which is very conducive to the charge storage of materials. All of these contribute to improving the electrochemical performance of the obtained MWC so that it has better energy storage capacity [46].

**Figure 4:**
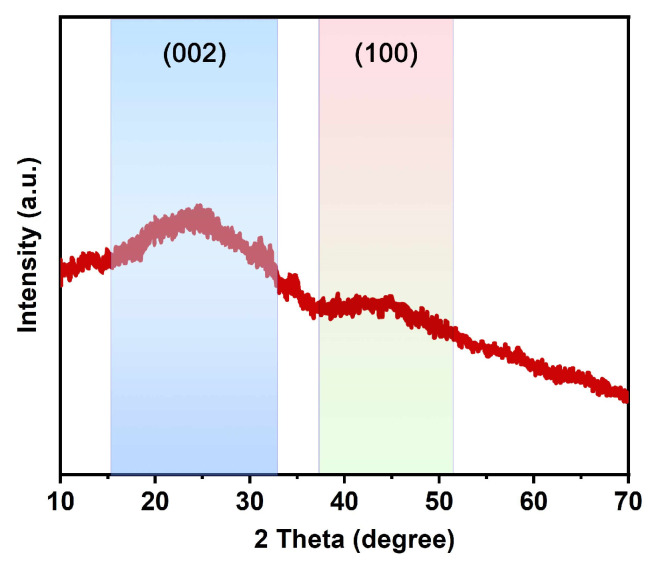
XRD patterns for MWC.

Figure 4 illustrates XRD spectrum of the MWC sample, revealing the carbon accumulation structure. At 23° is the (002) plane and at 46° is the (100) plane showing an amorphous graphite framework of MWC. Additionally, the (002) plane shows an offset from 26.4° of graphite to a lower 23.4–24.7°, indicating expansion of interlayer spacing [47]. This can be attributed to the assimilation of phosphorus at the edge of the carbon layer, which causes the deformation of the graphite plane and increases the interlayer spacing [48]. In addition, (002) and (100) of MWC show wide diffraction peaks, indicating that additional heteroatomic doping will induce more carbon structure defect sites and disturbances, providing more active sites for electrochemical reactions [49].

**Figure 5:**
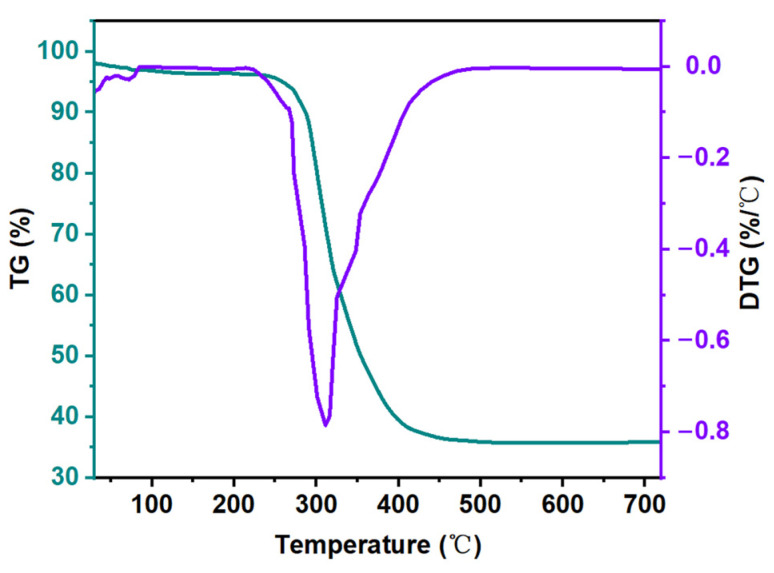
TG and DTG diagrams of MWC.

The decomposition of watermelon peels and watermelon peel carbon material in air were analyzed by TGA, and the TGA curve is shown in Figure 5. The first is evaporation of water and volatile materials, and the carbon material presents a slight weight loss [50]. After that, the watermelon peel carbon material sustained rapid weight loss at 230 °C to 402 °C. At this temperature, the watermelon biomass degraded, showing 65% weight loss. The second weight loss platform could be attributed to the break of carbon-carbon bond, at which time MWC was almost completely carbonized into carbon in the air [51]. Higher hydrothermal temperature may also lead to secondary degradation of a hydrate in the carbonization process, resulting in lower hydrate production [52]. Finally, after 511 °C, the residual carbon rate was only 34%. The DTG image of MWC (Figure 5), as the derivative of the temperature function, shows the change of the measured mass with the rate of temperature decline. The characteristic peak of lignin appears at 309 °C, which is the typical peak of lignin [53]. Because lignin has a number of different heat-stabilized bonds, mass loss occurs over a wide temperature range [54].

### 4.2. Electrochemical Performance of MWC

**Figure 6:**
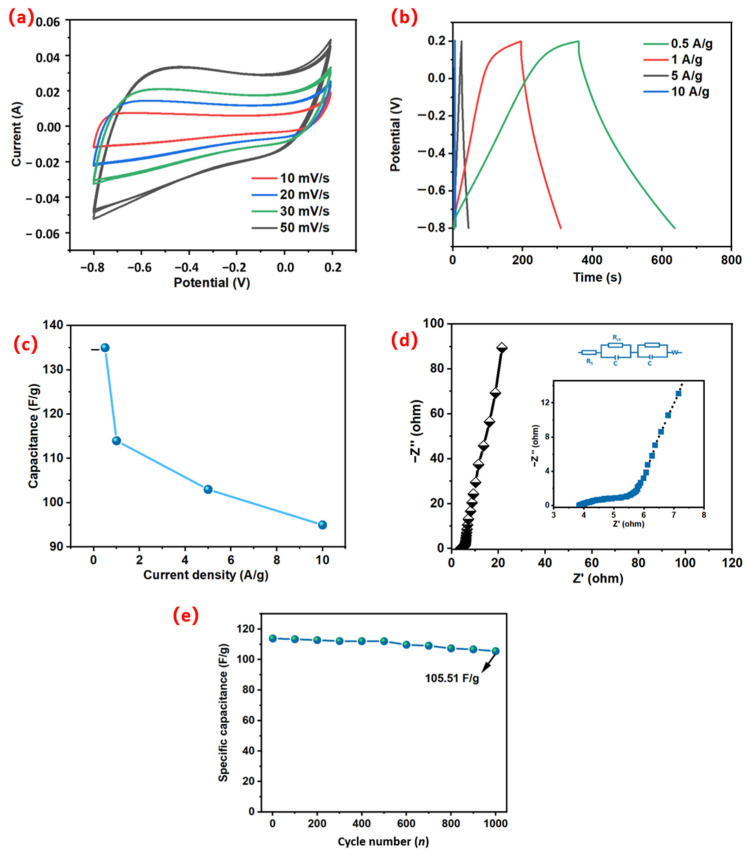
(**a**) CV curves of MWC at various scan rates (10−50 mV/s) using a three-electrode system. (**b**) GCD curve for MWC at 0.5, 1, 5, 10 A/g. (**c**) The capacitance of the as-prepared electrodes in 6 M KOH at various current densities. (**d**) The Nyquist plots of MWC electrode in 6 M KOH media. (**e**) Cycle stability of MWC after 1000 cycles of charge/discharge under a current load of 1 A/g.

The CV and GCD were measured and analyzed to understand the electrochemical performance of watermelon carbon material. All GCD curves are linearly dependent on time and are approximately symmetrical triangles (Figure 6b). It presents a long discharge time and excellent electrochemical property. The specific capacitance is 135.2, 114.1, 103.5, and 95.2 F/g, respectively, when the current density is 0.5, 1, 5, 10 A/g (as shown in Figure 6c). The excellent porous structure, large pore volume, successful doping of heteroatoms, as well as activation of electrolyte KOH, result in excellent specific capacitance of MWC. Due to the lack of timely diffusion of electrolyte ions, the specific capacitance decreases after the current density increases [31]. Compared with other biomass supercapacitors, the specific capacitor has excellent performance (Table 1).

As shown in Figure 6a, the cyclic voltammetry curve (CV) of the working electrode was obtained at the sweep speed of 10, 20, 30, 50, and 100 mV/s. All CV curves are approximated as similar rectangles with slight changes in absolute area, with the largest area and maximum capacitance at 50 mV/s. When scanned at 50 mV/s, there was a distinct hump around −0.5V (Figure 6a). The presence of the hump is attributed to the REDOX reaction between the MWC and the nitrogen and oxygen functional groups in the electrolyte, which demonstrates the contribution of the pseudocapacitance to the total capacitance [55]. The scanning rate increased gradually, and the CV curve shape did not change significantly, indicating that the structure of MWC was conducive to mass storage and rapid transfer of charge [12]. The higher capacity may be due to the high ionization potential of KOH, and the activation gives the material a porous structure, leading to an increase in the specific surface area of the material and an increase in capacitance. At the same time, nitrogen, oxygen, or phosphorus doping causes the increase of pseudocapacitance, which enlarges the battery voltage of the supercapacitor [42].

In order to probe into the chemical and physical processes occurring in the electrodes, electrochemical impedance spectroscopy (EIS) was analyzed (Figure 6d). In the high-frequency region, MWC shows a distinct semicircle, indicating a strong interaction between the heteroatomic groups and the hydrogen ions [43]. Secondly, the low frequency region shows a linear slope, representing the Warburg impedance. The EIS curve of MWC electrode material is approximately a straight line with high slope, indicating a relatively low ion diffusion resistance. The main reason is that the porous structure of MWC electrode material is more favorable for electron penetration and transport, which is in accordance with the outcomes of CV and GCD. The EIS spectral fitting equivalent circuit is briefly presented in Figure 6d, where R_S_ is the resistance of the bulk cell (e.g., electrolyte and diaphragm), W is the resistance resulting from surface film passivation, and R_ct_ is the charge transfer resistance at the electrode/electrolyte interface [36]. As shown in Figure 6d, MWC has a low resistance, which indicates that the material has excellent ion diffusion and charge transfer ability.

The cycling stability of MWC was tested after 1000 charge/discharge cycles under a current load of 1 A/g (Figure 6e). Even after 1000 charge and discharge cycles, the specific capacitance remained at 105.51 F/g. As shown in Figure 2e,f, the SEM images show that the surface morphology of the MWC has not collapsed much after 1000 charge/discharge cycles. This shows its excellent cycling stability, indicating that this is an efficient supercapacitor electrode material with good prospects for development and utilization.

**Table 1:** Research progress of biomass electrode application in supercapacitors.

Biomass Precursor	Synthetic Method	Discharge Capacitance	Electrolyte	Current Density	Ref.
ZnO-AC nano-composite	sol-gel method	38.4 F/g	6 M KOH	2 mA/cm^2^	[56]
Chlorella	electrospun to nanofibrous material, carbonization and activation	182 F/g	1 M H_2_SO_4_	0.5 A/g	[57]
Potato	self-catalytic activation	54 F/g	6 M KOH	0.5 A/g	[58]
Carbon nanofibers derived from polyimide and lignin precursors	electrospining and carbonization.	83.6 F/g	6 M KOH	1 A/g	[59]
Waste cotton stalk	carbonization and activation	111.1 F/g	6 M KOH	1 A/g	[60]
Activated carbon	-	150.7 F/g	1 M Na_2_SO_4_	0.1 A/g	[61]
Pleurotus eryngii	carbonization and KOH activation	195 F/g	6 M KOH	0.2 A/g	[62]
Jackfruit peel	three-step approach consisting of KOH activation and CO_2_ activation at different temperatures	191 F/g	1 M H_2_SO_4_	-	[63]
Watermelon peel	one-step carbonization	135.2 F/g	6 M KOH	1 A/g	This work

## 5. Conclusions

In this paper, using cheap and abundant watermelon peels as raw material, we successfully synthesized nitrogen and phosphorus double-doped bio-based polyporous carbon by one-step carbonization method and explored its use as a renewable carbon source as a low-cost energy storage device. Using XPS, XRD, and other characterization methods, it can be found that nitrogen and phosphorus doping was successfully achieved in this preparation process. When the current density was 1 A/g, specific capacitance can be up to 135.2 F/g. Under a current load of 1 A/g, even after 1000 charge/discharge cycles, the specific capacitance remained at 105.51 F/g. In comparison with other similar supercapacitors, the MWC electrode based supercapacitor exhibits comparable or even better electrochemical performance, while this kind of carbon materials manufactured by this approach have the characteristics of cheap raw materials, simple preparation process, green sustainable, excellent electrochemical performance, etc., which will be very promising as electrode materials for supercapacitors and indicates the direction for the next exploration of efficient utilization of biomass waste.

## Data Availability

Not applicable.
